# A two-step inverse solution for a single dipole cardiac source

**DOI:** 10.3389/fphys.2023.1264690

**Published:** 2023-09-07

**Authors:** Beata Ondrusova, Peter Tino, Jana Svehlikova

**Affiliations:** ^1^ Institute of Measurement Science, Slovak Academy of Sciences, Bratislava, Slovakia; ^2^ Faculty of Electrical Engineering and Information Technology, Slovak University of Technology in Bratislava, Bratislava, Slovakia; ^3^ School of Computer Science, University of Birmingham, Birmingham, United Kingdom

**Keywords:** body surface potential mapping, inverse problem of electrocardiography, greedy algorithm, transfer matrix, premature ventricular contractions

## Abstract

**Introduction:** The inverse problem of electrocardiography noninvasively localizes the origin of undesired cardiac activity, such as a premature ventricular contraction (PVC), from potential recordings from multiple torso electrodes. However, the optimal number and placement of electrodes for an accurate solution of the inverse problem remain undetermined. This study presents a two-step inverse solution for a single dipole cardiac source, which investigates the significance of the torso electrodes on a patient-specific level. Furthermore, the impact of the significant electrodes on the accuracy of the inverse solution is studied.

**Methods:** Body surface potential recordings from 128 electrodes of 13 patients with PVCs and their corresponding homogeneous and inhomogeneous torso models were used. The inverse problem using a single dipole was solved in two steps: First, using information from all electrodes, and second, using a subset of electrodes sorted in descending order according to their significance estimated by a greedy algorithm. The significance of electrodes was computed for three criteria derived from the singular values of the transfer matrix that correspond to the inversely estimated origin of the PVC computed in the first step. The localization error (LE) was computed as the Euclidean distance between the ground truth and the inversely estimated origin of the PVC. The LE obtained using the 32 and 64 most significant electrodes was compared to the LE obtained when all 128 electrodes were used for the inverse solution.

**Results:** The average LE calculated for both torso models and using all 128 electrodes was 28.8 ± 11.9 mm. For the three tested criteria, the average LEs were 32.6 ± 19.9 mm, 29.6 ± 14.7 mm, and 28.8 ± 14.5 mm when 32 electrodes were used. When 64 electrodes were used, the average LEs were 30.1 ± 16.8 mm, 29.4 ± 12.0 mm, and 29.5 ± 12.6 mm.

**Conclusion:** The study found inter-patient variability in the significance of torso electrodes and demonstrated that an accurate localization by the inverse solution with a single dipole could be achieved using a carefully selected reduced number of electrodes.

## 1 Introduction

The inverse problem of electrocardiography (ECG), i.e., Electrocardiographic Imaging (ECGI) is a non-invasive method that involves the use of body surface potentials (BSPs) and a patient-specific geometrical model of the torso to study the heart’s function (Gulrajani, 1998). One of the outcomes of the inverse solution is an identification of the location of the ectopic activity, such as premature ventricular contractions (PVCs) (Wissner et al., 2017; Svehlikova et al., 2018), Wolff-Parkinson White syndrome (Ghosh et al., 2008), myocardial scar (Horáček et al., 2015), etc. This information can be used to guide the cardiologist to the site of undesired activity before a radiofrequency catheter ablation (RFA) (Parreira et al., 2020a).

Various multi-lead ECG systems, such as those with 64 (Oosterhoff et al., 2016), 128 (Kadanec et al., 2017), 192 (Bergquist et al., 2020), and more electrodes (Yu et al., 2018) are employed to capture body surface potentials during body surface potential mapping (BSPM). The density of BSP recordings on the anterior part of the torso is usually higher in multi-lead ECG systems with fewer electrodes [e.g., 64 (Roudijk et al., 2021)], while systems with more electrodes [e.g., 128 (Kadanec et al., 2017)] distribute the electrodes on the anterior and posterior sites more uniformly. Several studies have demonstrated that approximately 30 electrodes are sufficient to gather adequate information regarding the heart’s electrical activity during BSPM (Barr et al., 1971; Lux et al., 1978; Finlay et al., 2006). The unanswered question is whether this would also be enough for the solution of the inverse problem.

In general, using more electrodes on the torso surface can provide more information about the electrical activity of the heart. Therefore, various electrode systems with a higher number of electrodes are used for solving the inverse problem (Sapp et al., 2012; Vijayakumar et al., 2014; Bear et al., 2019). In clinical practice, it is often difficult to attach all electrodes of a multi-lead ECG measuring system (e.g., due to a local skin problem), or some electrodes need to be excluded from the inverse solution (e.g., due to bad contact) (Parreira et al., 2020b). Fortunately, signals across the electrodes naturally exhibit dependencies and indeed, it has been demonstrated that the inverse problem can be accurately solved using a smaller number of electrodes without the need to employ all electrodes; however, the optimal number required varies among studies. As an example, Gharbalchi No et al. (2020) used a sequential approach for the selection of the torso electrodes. The electrode sets leading to the highest agreement between the reconstructed and recorded epicardial potentials were used. The study showed that using 32 electrodes out of 192 electrodes could lead to an inaccurate solution, while 64 electrodes provided similar accuracy to using all 192 electrodes. The reduced accuracy of the inverse solution with 32 electrodes could be attributed to the fact that these electrodes were situated anteriorly. In contrast, when using 64 electrodes, both anterior and posterior electrodes were used, leading to improved results. Similarly, Parreira et al. (2020b) suggested that a larger number of electrodes should be used to achieve a good spatial resolution in patients with PVCs. The researchers compared non-invasive maps computed by the solution of the inverse problem using different configurations of electrode bands with the maps recorded during invasive electroanatomic mapping. The study revealed that using 74 electrodes resulted in a good spatial resolution compared to using 38 electrodes. In both cases, the electrodes were positioned anteriorly as well as posteriorly. While other studies also investigate the ideal number and arrangement of electrodes, we have specifically highlighted the studies mentioned above due to their recent publication dates and their alignment with the objective of our study.

This study aims to explore the positions of the most significant electrodes and analyze how they influence the accuracy of the inverse solution to a single dipole. To meet this objective, a two-step inverse solution is proposed. The proposed two-step inverse solution is a novel computationally undemanding approach allowing us to recompute the inverse solution using only the most significant electrodes. The significance of the electrodes is investigated from the singular values obtained by the singular value decomposition (SVD) of the transfer matrix, which contains information about the geometrical and electrical properties of the torso. This approach differs from the previously proposed approach using SVD (Dossel et al., 2002) since the significance of the electrodes is estimated from the transfer matrix corresponding to the preliminary estimated origin of the PVC assuming a single dipole cardiac source. During the implementation, two assumptions have been made: 1) that the positions of the most significant electrodes depend on the origin of the cardiac activity (Ondrusova et al., 2021) and 2) the significance of the torso electrodes would remain similar for small perturbations of the origin (Ondrusova et al., 2023). Thus, in the first step of the proposed approach, all electrodes are used to compute the first-step inverse solution to approximately locate the source of the cardiac activity. This is then used to determine the electrode significance. Second, the inverse solution is recomputed starting with the four most significant electrodes detected in the first step. Further electrodes are then added one by one according to their decreasing significance values. This enables us to concentrate on the electrodes that provide the most significant information and exclude the irrelevant ones from the inverse solution. Using irrelevant electrodes can lead to solutions with increased susceptibility to errors*.* The localization error of the results for each number of electrodes was evaluated.

## 2 Materials and methods

### 2.1 Bratislava data

In total, 13 patients (10 male, 3 female, 52 ± 17 years old) with spontaneous PVCs underwent BSPM with the multi-lead measuring system ProCardio8 (Kadanec et al., 2017) that uses 128 torso and 4 limb electrodes. The 128 torso electrodes were evenly placed on the patient’s torso. The electrodes are built within self-adhesive strips; each strip contains 8 electrodes so 16 vertical strips were used. Further, 3 limb electrodes were placed on the left and right arm and left leg. All recorded signals were measured against the common-sense electrode (CMS) electrode. A driven right leg (DRL) circuit using the electrode on the right leg was used to suppress common mode voltage (Kadanec et al., 2017). The measurement of the heart’s activity took from 5 to 20 min with a sampling frequency of 1000 Hz. After the BSPM, the position of the torso electrodes and geometrical properties of the patient’s torso were captured using a Toshiba Aquilion One CT scan. The scanning was synchronized with the patient’s cardiac cycle.

The measurements were done in collaboration with the National Institute of Cardiovascular Diseases (NICD) in Bratislava, Slovakia and approved by the Ethical Committee of NICD. The patients were informed about the potential risks of the measurements. The informed consent was signed by those who decided to participate in this study. All measurements were done following the Good Clinical Practice guidelines and Helsinki Declaration for Biomedical Research. More details about the Bratislava data can be found in (Svehlikova et al., 2022).

Measured patients in the range of days to months underwent the RFA procedure in order to localize and destroy the origin of their PVCs as shown in Table 1. The information from the RFA procedure was used as the ground truth for the evaluation of the accuracy of the proposed method.

**TABLE 1:** The information for the Bratislava data.

ID	P001	P002	P004	P006	P008	P010	P020	P021	P023	P024	P027	P029	P036
Age [years]	17	63	72	56	46	59	37	33	38	79	57	64	61
Gender [-]	M	M	F	M	M	M	M	M	M	M	F	F	M
Time between BSPM and RFA [days]	178	282	5	350	2	331	40	61	384	8	425	0	157

#### 2.1.1 Data processing

The CT scans were used to create a patient-specific geometrical model of the torso, ventricles (endo-epicardial model), right and left lung lobe and right and left blood cavities in the form of 3D triangulated meshes. In total, 6 geometrical models were created using semi-automatic segmentation methods in the software TOMOCON provided by Tatramed company (https://tatramed.sk/tomocon-workstation/) for each patient.

All signals measured by the multi-lead ECG measuring system ProCardio8 were pre-processed. To eliminate baseline drift, a high-pass filter (Blackmann-Harris window) was applied with a cutoff frequency of 0.5 Hz. The QRS complexes were detected using the Pan-Tompkins algorithm (Pan and Tompkins, 1985). The *k*-means algorithm (Christopher, 2006) was utilized to cluster all QRS complexes between the ones associated with the sinus beats and the ones corresponding to the ectopic beats (PVCs). The ectopic beats were then averaged in each lead and the BSP map $\Phi_{B}\in R^{128\times 30}$ was calculated for each patient, covering the first 30 ms of the cycle. The applied signal processing methods are described in more detail in (Svehlikova et al., 2022).

### 2.2 A two-step inverse solution

**FIGURE 1:**
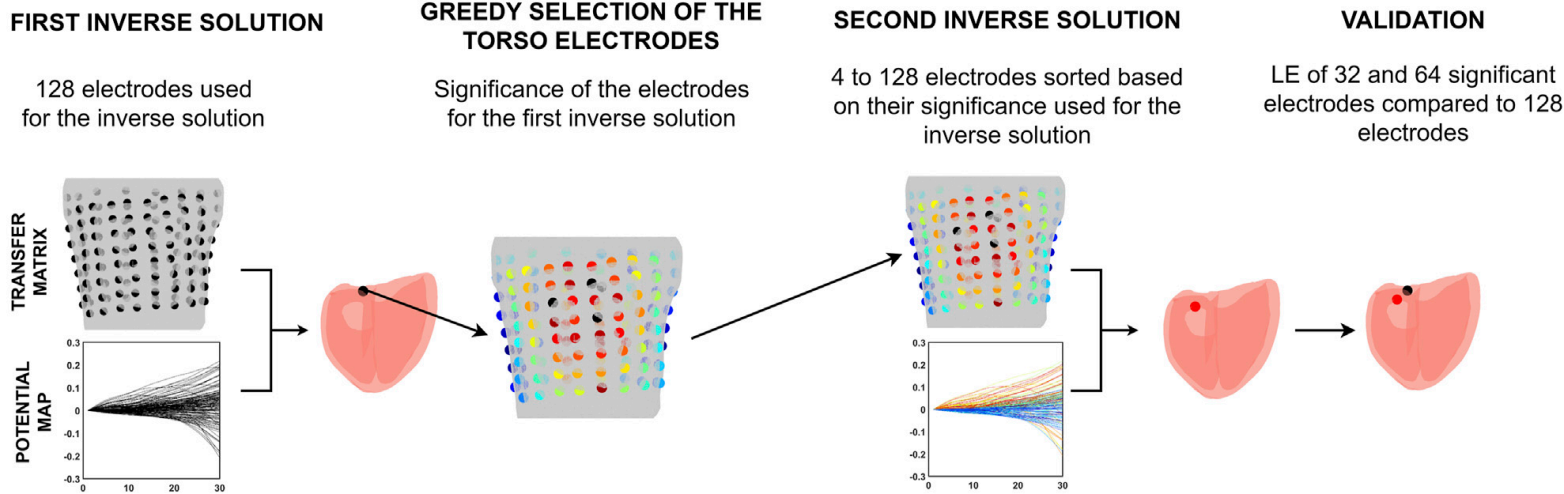
The two-step inverse solution pipeline.

This study presents a novel two-step approach for solving the inverse problem, intending to use only the most significant torso electrodes. Figure 1 illustrates the pipeline of this two-step inverse solution.

First, the inverse problem with a single dipole for localization of the PVC was solved using information from all electrodes. Then, the significance of the electrodes was computed for the position of the single dipole corresponding to this first-step inverse solution by a greedy algorithm. Second, the inverse problem was solved using electrodes sorted according to their significance in descending order. The accuracy of the inverse solution was assessed using the 32 and 64 most significant electrodes as used in (Finlay et al., 2006; Gharbalchi No et al., 2020) and compared to the accuracy of the inverse solution when all electrodes were used.

#### 2.2.1 First inverse solution

The first inverse solution was computed using information from all torso electrodes according to the equation

$$S_{C}=T^{+}\Phi_{B},$$ (1)

where $S_{c}$ is the cardiac source, $T^{+}$ is the Moore-Penrose pseudoinverse of the transfer matrix T and $\Phi_{B}$ is the BSP map. The cardiac source $S_{c}$ is represented by a single dipole based on the assumption that the activated area of the myocardium during the onset of the PVC (up to 30 ms) is sufficiently small to be accurately described by a single dipole. The transfer matrix T is used to compute potentials on the body surface given the known position of the cardiac electrical source (a single dipole). While a single dipole cardiac source maintains a constant position, its magnitude and orientation may vary. This results in a transfer matrix where each column aligns with one of the 3 dipole’s orthogonal components, representing the orientation of the dipole. These components are situated along the mutually perpendicular X, Y, and Z-axes. The transfer matrix takes into account the geometry and conductivity properties of the volume conductor and the location of the electrodes on the body surface. Assuming 128 torso electrodes, the size of the transfer matrix for a single dipole position is thus $T\in R^{128\times 3}.$ The transfer matrix T was computed using the Boundary Element Method (BEM) (Barr et al., 1966; Geselowitz, 1967) for each patient and the homogeneous and inhomogeneous model of the volume conductor. The inhomogeneous model comprises structures with distinct conductivities, such as lungs filled with air (0.25 times the torso conductivity) and heart cavities filled with blood (3 times the torso conductivity). The inverse problem in the form of dipole moments was solved for all predefined positions of the cardiac source $S_{c}$ at each time instant. The predefined source positions were restricted to the vertices of the endo-epicardial ventricular triangulated surface. For each position j of the cardiac source $S_{c}$ , the relative residual error (RRE) was computed in each time instant $t\in \langle 1,30\rangle$ as

$$RRE\left(t,j\right)=\frac{\sqrt{\Phi_{B}^{2}\left(t\right)-\Phi_{C}^{2}\left(t,j\right)}}{\sqrt{\Phi_{B}^{2}\left(t\right)}},$$ (2)

where $\Phi_{B}$ refers to the measured map and $\Phi_{C}$ is the map computed by a single dipole (Svehlikova et al., 2022). A smaller RRE indicates a closer agreement between the measured map $\Phi_{B}$ and the map computed from a single dipole $\Phi_{C}$ , and thus a better representation of the cardiac electrical activity. The best position of the inversely computed single dipole was chosen according to the criterion of minimum of $RRE\left(t,j\right)$ for all positions *j* and time instants *t*.

#### 2.2.2 Greedy selection of the significant torso electrodes

The output of the first inverse solution is the position (cartesian coordinates) and orientation (dipole moments) of a single dipole on the heart’s mesh that best describes the electrical activity of the PVC in its earliest phase. For this position, the significance of the electrodes was estimated from the singular values obtained from the SVD of the transfer matrix. The average localization error of inverse solution using a single dipole cardiac source is 20–30 mm (Svehlikova et al., 2018; Dogrusoz et al., 2023). Therefore, we assumed that the significance of the electrodes for the position of the first inverse solution would be similar to the significance of the electrodes for the position of the ground truth if these two positions are not too far apart (Ondrusova et al., 2023).

Let T be the transfer matrix with size $T\in R^{128\times 3}$ corresponding to the inversely computed position of the PVC origin. The matrix T can be decomposed using SVD as

T=UΣV. (3)

The matrices U and V are orthogonal basis matrices and the matrix Σ contains at most 3 non-zero singular values $\sigma_{1}\geq \sigma_{2}\geq \sigma_{3}$ on its diagonal. In the following, we assume that all three significant singular values are non-zero, which indeed was the case in all our experiments. T as a linear operator embeds a 3-dimensional unit ball into the 128-dimensional space in the form of a 3-dimensional ellipsoid living in $R^{128}$ . The lengths of the principal semi-axis of this ellipsoid are equal to the three singular values $\sigma_{1,}\sigma_{2},\sigma_{3}$ . Here, the singular values carry information about the significance of the basis in the electrode space (columns of U ) and cardiac/dipole source space (columns of V ). Three criteria were tested for the selection of the electrodes. Criterion A minimizes the conditioning number as:

$$A=\min\left(\frac{\sigma_{1}}{\sigma_{3}}\right).$$ (4)

This criterion measures the amount of distortion the unit ball undertakes when transformed by T .

Criterion B maximizes the product of the singular values (volume of the image of the unit ball under the linear operator T ):

$$B=\max\left(\sigma_{1}{\;∙\;\sigma }_{2}∙\sigma_{3}\right).$$ (5)

Criterion C aims to maximize the total variation represented by the sum of the singular values:

$$C=\max\left(\sigma_{1}\;{+\;\sigma }_{2}+\sigma_{3}\right).$$ (6)

Criterion C is called the nuclear norm of the matrix that is a generalization of the matrix’s rank, which is a measure of its dimensionality.

**FIGURE 2:**
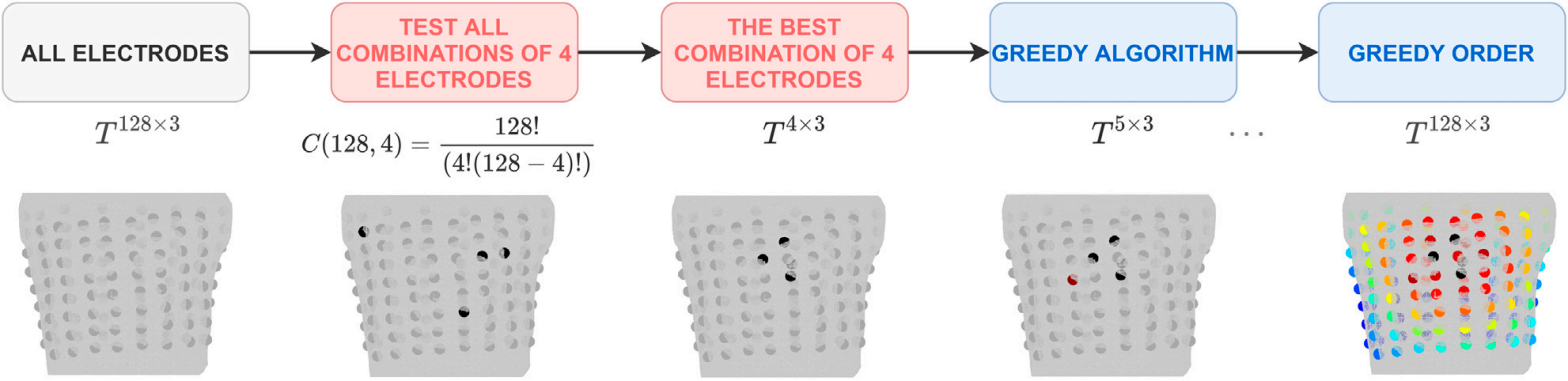
The pipeline for the greedy selection of the significant electrodes. The greedy order was determined separately for each criterion. It is important to note that the electrodes within the starting combination of 4 electrodes might have a posterior localization and this pipeline’s visualization is limited to the anterior view.

In order to identify the combination of electrodes that best fulfilled the given criteria, a greedy selection approach was implemented as shown in Figure 2. A greedy algorithm is an optimization approach that locally selects the best option at each step, without considering the potential impact on future steps. Therefore, only a locally optimal solution is chosen in each step. Greedy algorithms are known for their ease of implementation and computational efficiency, but they do not always guarantee to find the globally optimal solution (Cormen et al., 2009). However, it would be computationally infeasible to test all combinations of electrodes and thus a greedy selection of the significant electrodes was implemented. The process for selecting torso electrodes using a greedy algorithm consisted of two tasks, each carried out for every criterion. First, the combination of four electrodes that best optimized the selected criterion was chosen from all possible combinations (a total of 10,668,000 combinations from 128 positions on the torso). Following this, the greedy selection of additional electrodes was applied. In each step of the greedy algorithm, one electrode was added (out of the remaining electrodes) to the previously selected set of electrodes so that the criterion was fulfilled as best as possible at that step. The greedy algorithm continued execution until all available electrodes were used. At the output, the electrodes were sorted in descending order according to their significance for the given criteria (hereafter referred to as the “greedy order”) (Ondrusova et al., 2022).

### 2.3 Validation

After the RFA procedure, the cardiologist manually marked ablation points from a successful RFA procedure on an endo-epicardial ventricular triangulated surface using procedure notes and data from intracardiac mapping systems. If multiple ablation points were marked, the mean ablation point was computed. The ablation point was assumed as the ground truth of the PVC origin.

The localization error (LE) was calculated as the distance between the inversely computed origin of the PVC and the ground truth using the Euclidean distance. The LE was computed for the inverse solution using all 128 electrodes and then using 4 up to 128 electrodes based on their greedy order.

To validate the two-step inverse solution, the LE obtained using 32 and 64 electrodes (Finlay et al., 2006; Gharbalchi No et al., 2020) sorted based on their significance was compared to the LE of the first solution using all 128 electrodes. The differences in LE between the second inverse solution using 32 or 64 electrodes and the first inverse solution are presented in millimeters (mm).

Additionally, the power of the measured BSPs in each electrode was computed as the square of its root mean square value (RMS). The orders of the electrodes according to their significance and power were compared.

## 3 Results

### 3.1 First inverse solution

The first inverse solution was computed using all electrodes and the results are summarized in Table 2. The average LE for all patients and the homogeneous torso model was 28.6 ± 12.1 mm and for the inhomogeneous torso 29.0 ± 12.1 mm. Overall, the LE was 28.8 ± 11.9 mm.

**TABLE 2:** Localization errors (mm) of the first inverse solution for homogeneous (HOM) and inhomogeneous (INH) torso models.

ID	P001	P002	P004	P006	P008	P010	P020	P021	P023	P024	P027	P029	P036
HOM	11.9	36.6	12.2	49.6	30.7	40.7	41.5	23.7	15.8	20.0	22.2	29.4	38.1
INH	13.4	36.6	13.1	47.7	32.3	32.8	36.0	21.2	22.1	18.2	24.0	27.9	52.1

### 3.2 Greedy selection of the torso electrodes

**FIGURE 3:**
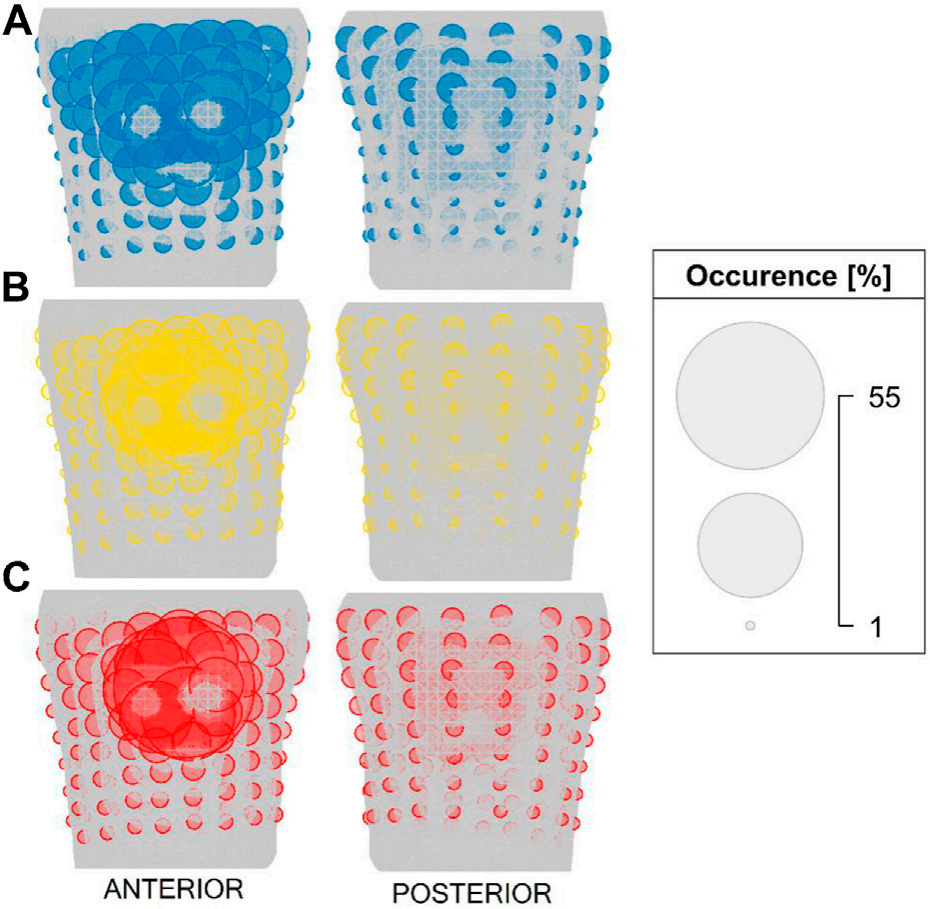
The positions of electrodes that occurred the most frequently within the top 1% of combinations of 4 electrodes for patient P001 and for criteria **(A)** (top), **(B)** (middle) and **(C)** (bottom). The size of the electrode corresponds to its occurrence.

**FIGURE 4:**
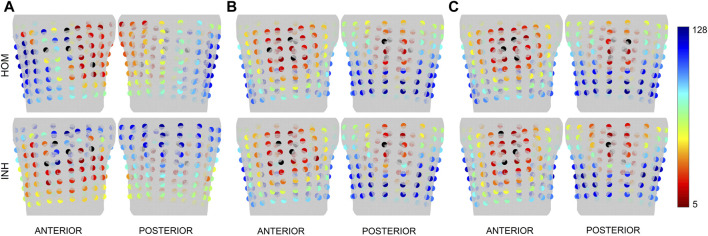
The greedy order of the electrodes for patient P001 depicted for the first inverse solution and the homogeneous (HOM) and inhomogeneous (INH) torso model and criteria **(A)** (left), **(B)** (middle) and **(C)** (right). The starting combination of 4 electrodes is represented in black, while the greedy order of electrodes from 5 to 128 is shown in spectra ranging from red to blue.

The significance of the torso electrodes was computed for the inversely estimated origin of the PVC (position of the first inverse solution). The electrodes are chosen gradually starting with 4 electrodes. The starting combination of 4 electrodes is selected from all possible combinations of electrodes (10,668,000 combinations). Figure 3 shows the positions of the electrodes that occurred the most frequently within the top 1% of combinations of 4 electrodes (106,680 combinations) for patient P001. It can be noticed that some electrodes were repeatedly selected and that the top combinations of 4 electrodes are localized within a specific region, predominantly comprising neighboring electrodes. Next, the greedy selection was applied. The greedy order of electrodes for patient P001 is presented in Figure 4, with the starting combination of 4 electrodes shown in black. The most significant electrodes are colored in red, while the least significant ones are colored in blue. The greedy order of electrodes was consistent for criteria B and C and both torso models, but criterion A had a different greedy order compared to criteria B and C. Additionally, the greedy order for criterion A varied between homogeneous and inhomogeneous torso models, respectively.

**FIGURE 5:**
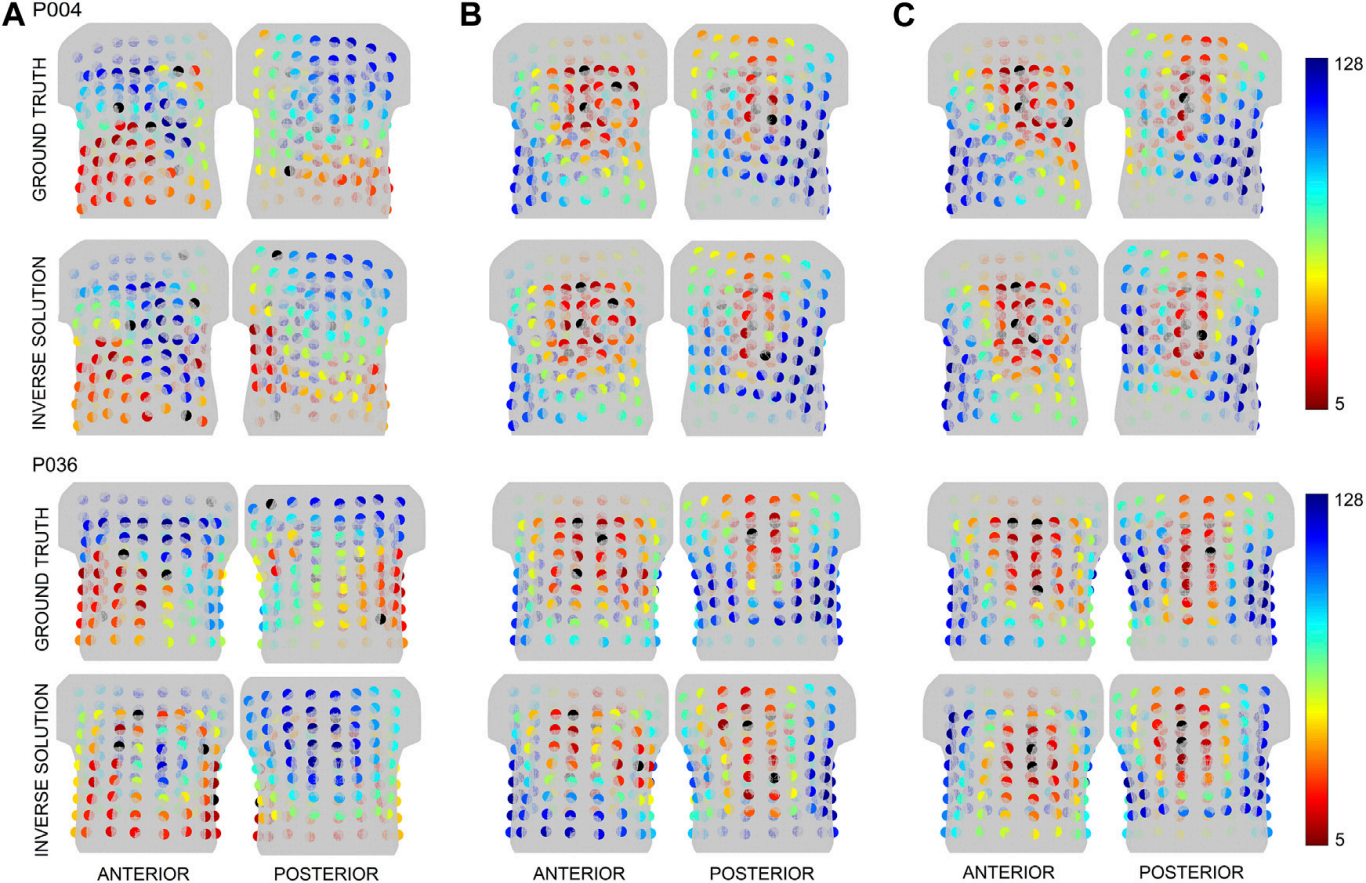
The greedy order of the electrodes for the patients P004 and P036 depicted for the ground truth and first inverse solution for the inhomogeneous torso model and criteria **(A)** (left), **(B)** (middle) and **(C)** (right). The starting combination of 4 electrodes is represented in black, while the greedy order of electrodes from 5 to 128 is shown in spectra ranging from red to blue.

This method assumes that a similar greedy order of the electrodes would be identified for ground truth and the inversely computed origin of the PVC if the distance between those two sites is small enough (Ondrusova et al., 2023). Figure 5 shows the greedy order of electrodes calculated for the ground truth and the first inverse solution for patients P004 (smallest LE) and P036 (highest LE) with an inhomogeneous model, using the same color representation as in Figure 4. For patient P004, the areas where the most significant electrodes were detected were similar between the ground truth and the first inverse solution for all criteria. For P036, a difference in the greedy order of electrodes was observed between the ground truth and the first inverse solution for all criteria mainly on the posterior side of the torso.

**FIGURE 6:**
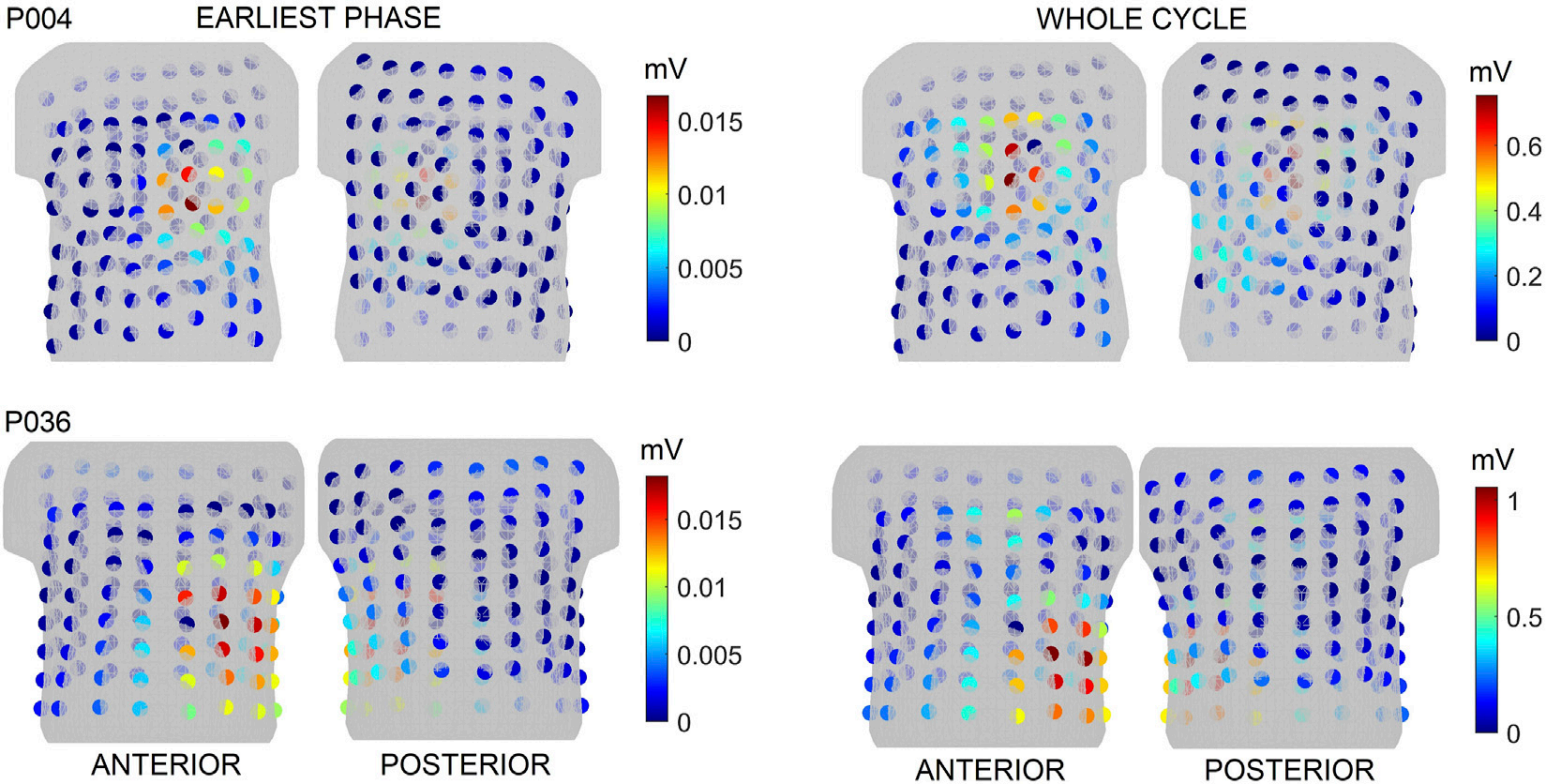
The power of the signal in each electrode in the earliest phase of the cycle and for the whole cycle for patients P004 and P036. The colorbar represents power values, where the spectra range from blue to red, with blue indicating low values and red indicating high values.

The greedy selection algorithm does not take into account recorded signals, which are time varying. The power of the signal in each electrode during the earliest phase (up to 30 ms–considering the inverse solution using a single dipole) and the whole QRS time interval are shown in Figure 6 for patients P004 and P036. For patient P004, the electrodes with the highest signal power and the highest significance computed for the ground truth for criteria B and C were found in the same anterior cranial plane. Interestingly, it can be observed that the positions of the most significant electrodes for the ground truth do not align with the positions of the electrodes with the highest power for patient P036.

### 3.3 Second inverse solution

**FIGURE 7:**
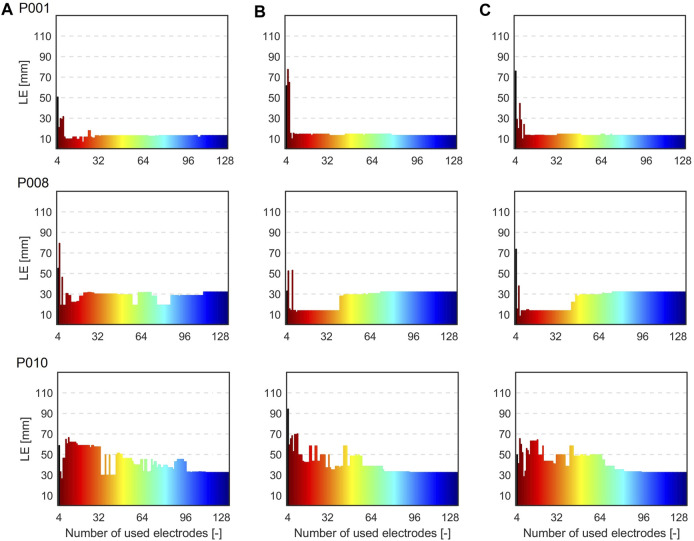
Localization errors (mm) when 4 to 128 electrodes based on their greedy order were used for the inverse solutions for the inhomogeneous torso model and patients P001, P008, and P010 and criteria **(A)** (left), **(B)** (middle) and **(C)** (right). The color of the bars indicates the range of electrodes used, e.g., green representing the inverse solution computed with 4 up to 64 electrodes based on their greedy order.

The second inverse solution was computed using electrodes according to their greedy order. A similar pattern of LE changes was identified across all patients. Specifically, when a limited number of electrodes was used, it resulted in inaccurate localization while using a larger number of electrodes led to increased localization stability. As a result, three distinctive cases were selected for the illustration. Figure 7 shows the LE obtained using from 4 to 128 electrodes based on their greedy order for the inhomogeneous model and patients P001, P008 and P010. The color of the bar indicates the range of electrodes used, for example, green color represents the inverse solution computed with 4 up to 64 electrodes, with the 64th electrode shown in green e.g., illustrated in Figure 4 for patient P001. The results indicate that a smaller number of significant electrodes can yield similar (P001, criteria A-C) or even better accuracy (P008, criteria B-C) compared to using all electrodes. However, in some cases, it may result in deterioration of localization (P010, criteria A-C).

**FIGURE 8:**
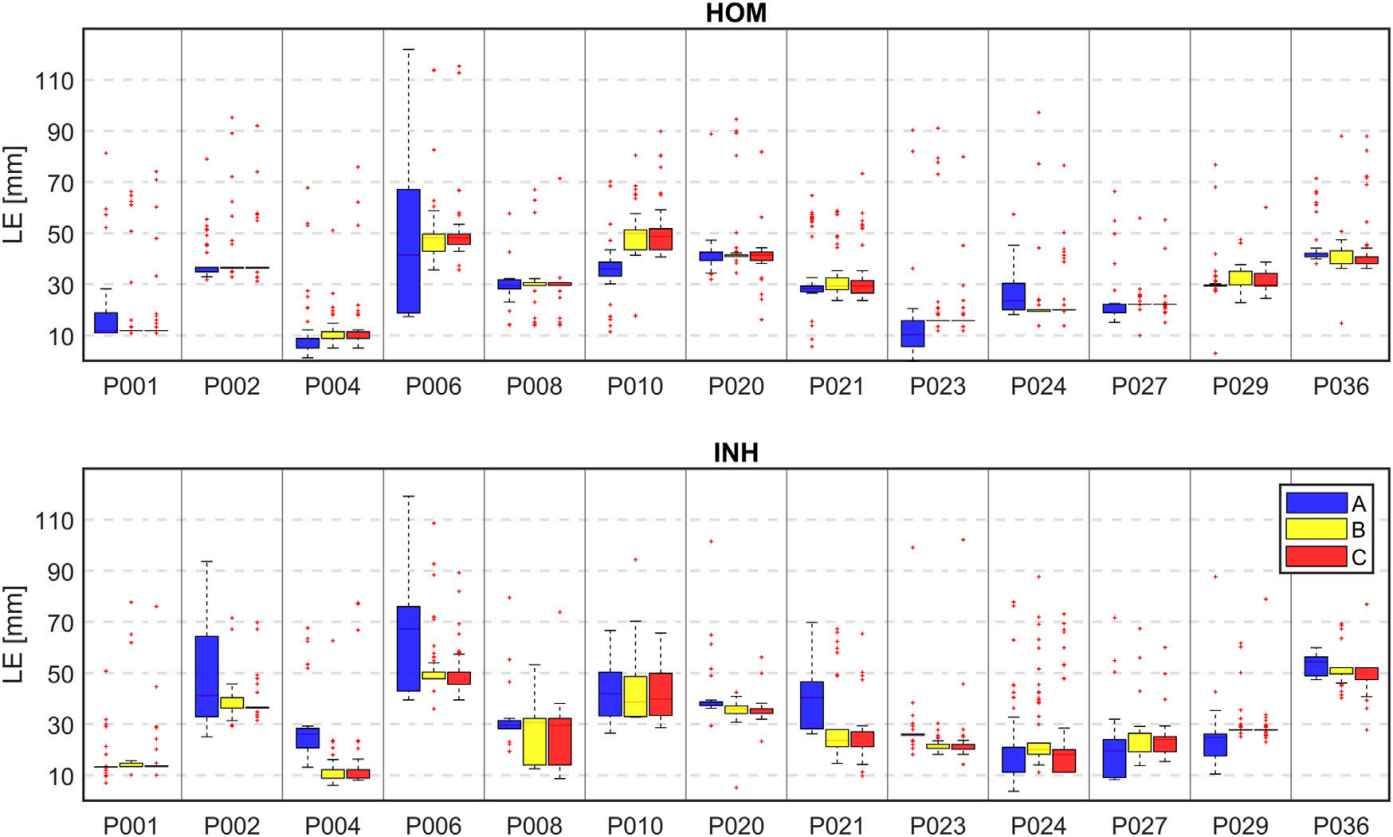
The boxplot of localization errors (mm) when 4 to 128 electrodes based on their greedy order were used for the inverse solutions for all patients, all 3 criteria (A–blue, B–yellow, C–red) and homogeneous (HOM) and inhomogeneous (INH) torso models.

Figure 8 displays boxplots of the LE values for all patients, both models and all criteria. Each box in the plot represents the statistical distribution of the LE values obtained using 4 to 128 electrodes for the inverse solution. The boxplots' outliers primarily correspond to the LEs obtained using a limited number of electrodes. The height of the boxes offers insights into the dispersion of the LEs. Smaller boxes indicate cases where there was minimal variance among the LE values, as seen in the case of patient P001 for the inhomogeneous model and all criteria. On the contrary, in certain cases, there was greater fluctuation in LE values (resulting in larger boxes), such as with patient P006 for both models and criterion A.

**FIGURE 9:**
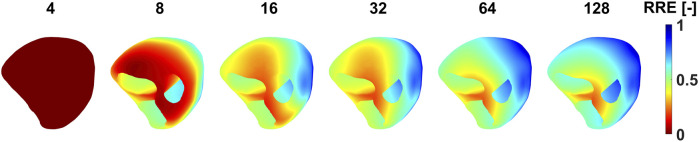
The distribution of RRE values on the endo-epicardial ventricular triangulated surface when a specific number of electrodes were used for the inverse solution. The RRE values are depicted for patient P001, criteria B and the homogeneous torso model.

The RRE values on the heart’s mesh as shown in Figure 9 were analyzed in order to investigate the stability and localization of the criterion for minimal RRE. The RRE value is assigned to each node which corresponds to a single dipole position. The node with the lowest RRE represents the winning node i.e., the node describing the electrical activity the best. With 4-8 electrodes, the node with the lowest RRE cannot be identified, because the RRE values are similar for all possible dipole positions on the heart. With more electrodes, it's easier to select the node with the lowest RRE from a localized area of low RRE values.

Table 3 shows the LEs when 32 and 64 electrodes sorted according to their significance were used for the inverse solution. When using 32 electrodes for the inverse solutions using the homogeneous torso model, improvement in localization accuracy was observed for criterion A (26.4 ± 15.8 mm), in contrast to the using all electrodes (28.6 ± 12.1 mm). However, there was a slight decline in performance for criteria B and C (30.0 ± 14.0 mm and 30.8 ± 14.9 mm, respectively) compared to using all electrodes. Interestingly, when considering the inhomogeneous model, a degradation in performance was observed for criterion A (38.8 ± 22.2 mm) compared to the use of all electrodes (29.0 ± 12.1 mm). Improvement was observed for criterion C (26.8 ± 14.4 mm) while similar accuracy was observed for criterion B (29.2 ± 15.9 mm). When using 64 electrodes for the inverse solutions and the homogeneous torso model, a slight improvement in localization was observed for criterion A (28.3 ± 16.1 mm) while slight worsening compared to using all electrodes (28.6 ± 12.1 mm) were observed for criteria B and C (29.5 ± 12.6 mm and 29.6 ± 13.2 mm). In contrast, there was a degradation in LE observed for criterion A and the inhomogeneous model (32.0 ± 17.9 mm), whereas comparable accuracy was noted for criterion B (29.3 ± 12.0 mm) and C (29.3 ± 12.5 mm) when compared to the use of all electrodes (29.0 ± 12.1 mm). In summary, the average LEs of the second inverse solutions were 32.6 ± 19.9 mm (A), 29.6 ± 14.7 mm (B), and 28.8 ± 14.5 mm (C) when 32 electrodes were used for both torso models. When 64 electrodes were used, the average LEs were 30.1 ± 16.8 mm (A), 29.4 ± 12.0 mm (B), and 29.5 ± 12.6 mm (C). The average LE using all electrodes was 28.8 ± 11.9 mm.

**TABLE 3:** Localization errors (mm) expressed as mean ± standard deviation when 32, 64, and 128 electrodes were used for the inverse solution for all 3 criteria and homogeneous (HOM) and inhomogeneous (INH) torso models.

	32 electrodes		64 electrodes		128 electrodes	
Criterion	HOM	INH	HOM	INH	HOM	INH
A	26.4 ± 15.8	38.8 ± 22.2	28.3 ± 16.1	32.0 ± 17.9	28.6 ± 12.1	29.0 ± 12.1
B	30.0 ± 14.0	29.2 ± 15.9	29.5 ± 12.6	29.3 ± 12.0	28.6 ± 12.1	29.0 ± 12.1
C	30.8 ± 14.9	26.8 ± 14.4	29.6 ± 13.2	29.3 ± 12.5	28.6 ± 12.1	29.0 ± 12.1

**FIGURE 10:**
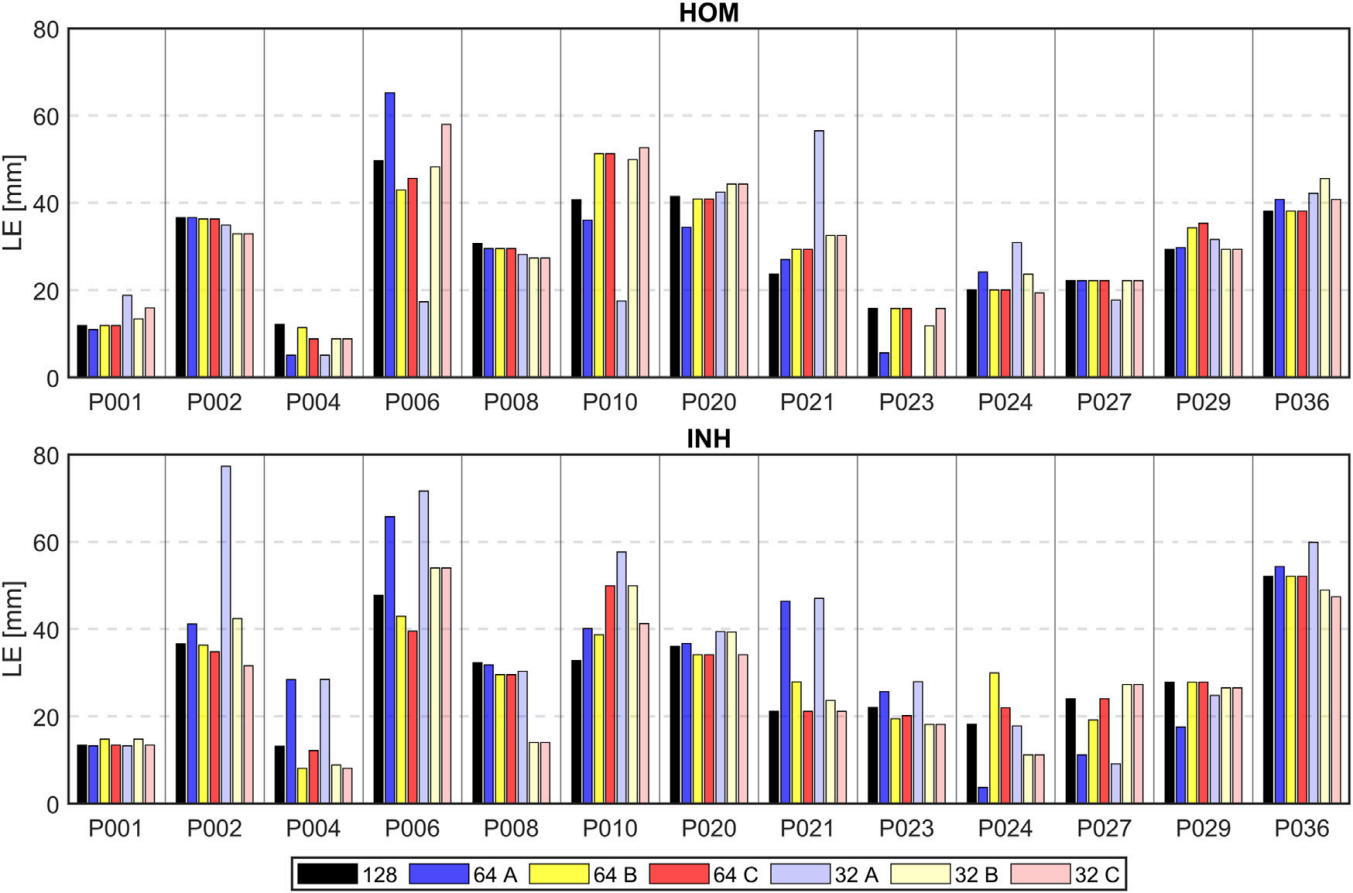
Localization errors (mm) of the inverse solution using 128 electrodes (depicted in black), 64 electrodes (depicted in darker colors), and 32 electrodes (depicted in lighter colors) sorted by their greedy order for all 3 criteria (A–blue, B–yellow, C–red) and homogeneous (HOM) and inhomogeneous (INH) torso models.

Lastly, the differences in LE were accessed between the second (32 and 64 electrodes used sorted based on their significance) and the first solution (all electrodes used) and displayed in mm. Positive values in Tables 4, 5 indicate higher localization errors, while negative values indicate improvement, and zero represents no change in the localization. Figure 10 illustrates the LEs obtained using 32 and 64 electrodes sorted based on their significance, as well as when all electrodes were used. These results are shown for all patients, both torso models, and all criteria.

**TABLE 4:** Differences in localization errors (mm) computed between the second (32 electrodes) and the first inverse solution (all electrodes) for all 3 criteria and homogeneous (HOM) and inhomogeneous (INH) torso models. Positive values indicate higher localization errors, negative values indicate an improvement in localization, and zero represents no change in localization.

HOM														
ID		P001	P002	P004	P006	P008	P010	P020	P021	P023	P024	P027	P029	P036
Criterion	A	6.9	−1.7	−7.1	−32.3	−2.5	−23.2	0.9	32.8	−15.8	10.9	−4.5	2.2	4.1
	B	1.6	−3.7	−3.3	−1.4	−3.3	9.2	2.8	8.9	−4.0	3.6	0.0	0.0	7.5
	C	4.1	−3.7	−3.3	8.3	−3.3	11.9	2.8	8.9	0.0	−0.7	0.0	0.0	2.7

**INH**														
ID		P001	P002	P004	P006	P008	P010	P020	P021	P023	P024	P027	P029	P036
Criterion	A	−0.2	40.7	15.3	23.9	−1.9	24.9	3.4	25.8	5.9	−0.3	−14.9	−3.0	7.8
	B	1.3	5.8	−4.3	6.2	−18.2	17.2	3.3	2.5	−3.9	−7.0	3.3	−1.3	−3.2
	C	0.0	−5.1	−5.1	6.2	−18.2	8.5	−1.9	0.0	−3.9	−7.0	3.3	−1.3	−4.7

**TABLE 5:** Differences in localization errors (mm) computed between the second (64 electrodes) and the first inverse solution (all electrodes) for all 3 criteria and homogeneous (HOM) and inhomogeneous (INH) torso models. Positive values indicate higher localization errors, negative values indicate an improvement in localization, and zero represents no change in localization.

HOM														
ID		P001	P002	P004	P006	P008	P010	P020	P021	P023	P024	P027	P029	P036
Criterion	A	−0.9	0.0	−7.1	15.6	−1.1	−4.7	−7.1	3.4	−10.2	4.1	0.0	0.4	2.7
	B	0.0	−0.3	−0.7	−6.7	−1.1	10.6	−0.6	5.7	0.0	0.0	0.0	4.9	0.0
	C	0.0	−0.3	−3.3	−4.0	−1.1	10.6	−0.6	5.7	0.0	0.0	0.0	5.9	0.0

INH														
ID		P001	P002	P004	P006	P008	P010	P020	P021	P023	P024	P027	P029	P036
Criterion	A	−0.2	4.5	15.3	18.0	−0.4	7.3	0.6	25.2	3.6	−14.5	−12.8	−10.3	2.2
	B	1.3	−0.3	−5.1	−4.8	−2.7	5.9	−1.9	6.7	−2.6	11.7	−4.8	0.0	0.0
	**C**	0.0	−1.8	−1.0	−8.3	−2.7	17.2	−1.9	0.0	−1.9	3.8	0.0	0.0	0.0

Additionally, the number of cases that resulted in improvement, worsening, or no change was calculated when 32 or 64 electrodes were used. Using 32 electrodes and the homogeneous torso model, localization improvement was observed in 7 cases out of 13 for criterion A, 5 cases for criterion B, and 4 cases for criterion C. Interestingly, deterioration was observed in 6 cases for all criteria (A, B, and C), while no change was observed in 2 cases for criterion B and 3 cases for criterion C. Using the inhomogeneous model, improvement in localization was observed in 5 cases for criterion A, 6 cases for criterion B and 8 cases for criterion C. Deterioration was observed in 8 cases for criterion A, 7 cases for criterion B and 3 cases for criterion C while no change was observed in 2 cases for criterion C. Using 64 electrodes and the homogeneous torso model, localization improvement was observed in 6 cases for criterion A and 5 cases for criteria B and C. Deterioration was observed in 5 cases for criterion A and 3 cases for criteria B and C while no change was observed in 2 cases for criterion A and 5 cases for criteria B and C. Using the inhomogeneous model, improvement was observed in 5 cases for criterion A, 7 cases for criterion B and 6 cases for criterion C. Deterioration was observed in 8 cases for criterion A, 4 cases for criterion B and 2 cases for criterion C and no change was observed in 2 cases for criterion B and 5 cases for criterion C.

Moreover, a summary was calculated for both volume conductors, presenting the percentage of cases exhibiting improvement, deterioration, or no change in localization. Using 32 electrodes, criteria A and C lead to improved localization in 46% and criterion B in 42% of cases. A degradation of localization error was observed in 54% (A), 50% (B), and 35% (C) of cases and no change was observed in 0% (A), 8% (B), and 19% (C) of cases. When considering both volume conductors and 64 electrodes, improvement in localization was observed in 42% (A), 46% (B), and 42% (C) of cases, while degradation was observed in 50% (A), 27% (B), and 19% (C) of cases, and no change was observed in 8% (A), 27% (B), and 38% (C) of cases.

## 4 Discussion

In this work, a two-step inverse solution assuming a single dipole cardiac source was implemented and the accuracy of the second inverse solution was investigated for 13 patients with spontaneous PVCs and homogeneous and inhomogeneous torso models.

The first inverse solution was computed using all 128 electrodes. Our results in Table 2 indicated that using a homogeneous volume conductor slightly improves the localization (28.6 ± 12.1 mm vs. 29.0 ± 12.1 mm) which is consistent with our previous findings reported for a single dipole cardiac source (Dogrusoz et al., 2023).

Then, the significance of the torso electrodes for each patient was estimated from the transfer matrix corresponding to the inversely computed origin of the PVC as it was shown that the positions of the most significant electrodes vary between patients, and they depend on the origin of the PVC (Ondrusova et al., 2021). Three distinct criteria were employed for the evaluation of the electrodes' significance. For each criterion, the proposed method initiates by selection of starting combination of 4 electrodes. Even though the starting combination of 4 electrodes might not represent the best choice, Figure 3 demonstrates that electrodes within the top 1% of combinations of 4 electrodes are situated within the identical region, encompassing neighboring electrodes. Then, the greedy order of electrodes was computed. As expected, similar significance profiles of electrodes were obtained for volume and total variation criteria B and C, respectively. The volume criterion would prove less useful if the operator T were non-injective (or close to being non-injective) with zero, or close-to-0 singular value $\sigma_{3}$ , even though the other singular values are significant. This, however, was not the case in our experiments. However, criterion A, which represents the conditioning number, neglects the second singular value, potentially missing a significant component of information transfer from the dipole space to the electrode space. Indeed, the significance of electrodes for criterion A differed from criteria B and C as shown in Figures 4, 5. Furthermore, for criterion A, notable differences were observed also between homogeneous and inhomogeneous torso models, as illustrated in Figure 4.

The two-step inverse solution assumes that the ground truth and the inversely estimated origin of the PVC would have a similar greedy order of electrodes if the Euclidean distance between these two locations is sufficiently small (Ondrusova et al., 2023) (e.g., 13.1 mm as shown in Figure 5 for patient P004). Conversely, differences in the greedy order would be observed if the Euclidean distance between the locations is large (e.g., 52.1 mm as shown in Figure 5 for patient P036). In this case, the ground truth was identified during RFA at the base of the left ventricle near the intraventricular septum, while the inversely estimated origin was localized at the posterior left ventricular free wall. As a result, the positions of the most significant electrodes differed mainly on the posterior side of the torso.

Figure 6 shows that the location of the most significant electrodes for criteria B and C coincided with the electrodes with the highest signal power for patient P004. However, this was not the case for patient P036. The results indicate the methodology based solely on the analysis of the transfer matrix provides new insights into the significance of torso electrodes, beyond what can be obtained from the analysis of measured signals alone. Furthermore, it is noteworthy that only a few electrodes exhibited high power, whereas the remaining electrodes displayed low power with similar amplitudes. Consequently, sorting the electrodes based on the power of the measured signals could pose a challenge.

All 13 patients within the Bratislava dataset underwent ablation either in the right ventricular septum and right ventricular outflow tract (11 patients) or in the left ventricular septum near the base of the ventricles (2 patients). Unfortunately, due to these similar regions of ground truth localization, a similar pattern of greedy order of electrodes was observed between all patients mainly for criteria B and C. Overall, in this paper, the greedy order of electrodes computed for the ground truth assuming inhomogeneous torso model was depicted for patients P004 and P036 in Figure 5. In the case of P004, the ground truth was localized anterolaterally in the right ventricular outflow tract, while for P036, it was identified anteriorly in the left ventricular septum at the level of the bicuspid valve. Thus, all patients shown in this paper had their ground truth localized in a similar region, specifically at the base of the ventricles near the septum. As a result, a corresponding pattern of greedy order of electrodes was observed among these depicted patients, with the most significant electrodes positioned anteriorly, and some electrodes identified posteriorly in the upper half of the torso. However, it's important to highlight that while there were similarities in greedy orders, the significance of electrodes is not identical across the various patients. Nevertheless, in our previous study (Ondrusova et al., 2023), it was demonstrated that when the ground truth was located in the apex of the right ventricle, the most significant electrodes were found anteriorly in contrast with the results shown in this study for the ground truths in the base of the ventricles.

The second inverse solution was computed using from 4 up to 128 electrodes sorted based on their significance. The best inverse solution was selected according to the criterion of the minimal RRE. However, as illustrated in Figure 9, no local minimum of RRE can be identified when too few electrodes (4-8 electrodes) is used for the inverse solution. Therefore, the inverse solutions obtained using a small number of electrodes should be interpreted carefully.

This study demonstrates that the inverse solution can be solved accurately without using all torso electrodes, which aligns with previous research findings (Parreira et al., 2020b; Gharbalchi No et al., 2020). It was shown that the localization does not change with the number of used electrodes as demonstrated in Figures 7, 8 for some patients. These findings align with previous studies (Svehlikova et al., 2020; Ondrusova et al., 2022) suggesting that the solution of the inverse problem using a single dipole cardiac source is a robust method and not significantly affected by the electrode omission. Tables 4, 5; Figure 10 depict a comparison between the LEs of the second inverse solution when 32 and 64 electrodes, sorted based on their significance were used and the LEs of the first inverse solution using all electrodes. It was shown that similar or better localization compared to using all electrodes was observed for criteria B and C which exhibit smaller differences and smaller standard deviations of differences compared to criterion A. Using 32 electrodes, the best performance in the improvement of localization was observed for criterion C (−0.1 ± 6.2 mm). Considering torso models, the inhomogeneous model and again criterion C produced better localization results (- 2.2 ± 6.7 mm) with 8 out of 13 cases that showed an improvement in LE. Using 64 electrodes yielded localization similar to that obtained using all electrodes as was shown in (Gharbalchi No et al., 2020). In 5 out of 13 cases, no change in the localization was observed for the homogeneous model and criterion B and C and the inhomogeneous model and criterion C. The findings presented in this study suggest that the solution of the inverse problem using a single dipole cardiac source is a robust method that remains unaffected even when signals from certain electrodes are omitted. Therefore, the two-step inverse solution could potentially help to validate the result of the first inverse solution. First, an approximate position of the inverse solution is calculated using all electrodes. Subsequently, a solution is computed using the significant electrodes (e.g., using 64 significant electrodes), and this solution could either coincide or deviate from the first inverse solution. When both the first and second inverse solutions are in agreement, it adds a higher level of reliability to the results. In contrast, if these two solutions significantly differ, it reduces the reliability of the results.

In this study, the greedy algorithm was implemented in order to investigate the significance of the torso electrodes. There is a resemblance between the sequential approach proposed by Gharbalchi No et al. (2020) and the greedy algorithm proposed in this work. In both cases, electrodes are incrementally added to the previously selected set in each step. However, it is important to highlight that our approach relies exclusively on the characteristics of the transfer matrix for electrode selection, whereas the sequential approach assesses the precision of the inverse solution itself. Additionally, the greedy order of electrodes was estimated for patients with PVCs. This stands in contrast to the mentioned study, wherein BSPs were computed by solving the forward problem using epicardial potentials acquired during ventricular pacing of a dog’s heart. As demonstrated, variations in ECGI accuracy exist between spontaneous PVCs and ventricular-paced beats (Bear et al., 2023). Additionally, the study omitted the patient-specific anatomical geometry, employing only a single homogeneous torso model. Contrary, the research by Parreira et al. (2020b) involved patients with PVCs with their specific geometries. However, the study did not incorporate a process for choosing specific torso electrodes; instead, it centered around the using of different electrode band configurations. Nonetheless, it is interesting to note that all these studies concluded that the inverse solution can be accurately solved with a reduced set of electrodes.

However, the present study has some limitations that should be noted. The ground truth was manually marked on the endo-epicardial ventricular triangulated surface by a cardiologist, which may introduce some level of uncertainty as the ground truth on the heart’s mesh may not necessarily correspond with the true origin of the PVC, and errors in terms of millimeters could occur. This could explain why a high LE was observed for some patients. In a prior study involving a single dipole cardiac source, we documented an LE of 24.5 ± 4.1 mm for the inverse solutions calculated using data with a clearly defined ground truth (Dogrusoz et al., 2023). Notably, this LE was lower and exhibited a more confined range of standard deviations in contrast to the current study’s results, which reported an LE of 28.6 ± 12.1 mm for the homogeneous volume model. Moreover, the outcomes of other methods indicated a lower LE, as demonstrated e.g., by Bear et al. (2023) and Zhou et al. (2019). This suggests that the manual transfer of the ground truth position to the ventricular mesh might affect the LE reported in this study. Furthermore, the accuracy of the proposed approach is limited by the accuracy of the inverse solution, as inadequate precision in the first step can significantly impact the outcomes of the second inverse solution since the significance of the torso electrodes is derived from the first inverse solution using all torso electrodes. Regrettably, the ill-posed nature of the inverse problem means that errors within the input data (transfer matrix and the BSPs) have the potential to be amplified in the output. However, it is important to note that the inverse solution with a single dipole cardiac source is well-regularized. The mean conditioning number of transfer matrices used for the first inverse solution was 1.3 ± 0.2. The small value of the conditioning number suggests that the transfer matrix is well invertible, indicating that small errors in the input data are unlikely to result in major errors in the output. Nevertheless, the transfer matrices were computed using patient-specific geometrical models created from the CT scans. Thus, inaccuracies in the segmentations of the torso and internal organs could arise due to their anatomical complexity as it was shown by Tate et al. (2022). This research shows that errors in organ segmentations, particularly those related to the heart, can impact the accuracy of the inverse solution (Gassa et al., 2022). Moreover, the precision of the inverse solution is impacted by presumptions of torso homogeneity or piece-wise homogeneity and isotropy during the calculation of the transfer matrix. Furthermore, the accuracy of the inverse solution is closely linked to the quality of the recorded BSPs. All the limitations could explain the cases where deterioration in localization was observed and should be studied more. Additionally, further investigation should be carried out on criteria B and C, as they have demonstrated more robust estimations of the significance of torso electrodes in comparison to criterion A and have yielded more stable results in terms of the localization of the origin of the PVC. Lastly, the objective of this research was not to decrease the number of torso electrodes used in BSPM. Instead, the aim was to explore whether all these electrodes are essential for achieving an accurate solution to the inverse problem. For a more thorough investigation into the optimal electrode placement, it would be necessary to carry out extensive studies involving various positions of cardiac sources and diverse electrode distributions for different patients.

## 5 Conclusion

In this work, an approach for the two-step inverse solution assuming a single dipole cardiac source was proposed. The two-step inverse solution was based on the evaluation of the significance of torso electrodes using the transfer matrix. Three different criteria for estimating the significance of the torso electrodes were tested, and it was found that the second and third criteria provided more stable and robust estimations of the significance than the first one. Additionally, it was demonstrated that the inverse solution could be solved accurately without using all torso electrodes. The results showed that using 32 significant electrodes for the inverse solution led to a more prominent improvement in localization compared to using 64 electrodes, particularly for the inhomogeneous torso model. However, using 64 electrodes lead to localization results similar to those obtained using all electrodes. These findings have implications for clinical practice as they suggest that the accuracy of the inverse solution’s localization may not be compromised by missing some electrodes and that using a smaller number of significant electrodes can improve the localization of the inverse solution.

## Data Availability

The raw data supporting the conclusions of this article will be made available by the authors, without undue reservation.
